# Organoselenium functionalized SBA-15 as a new catalyst for the cyanide-free conversion of oximes to nitriles

**DOI:** 10.1186/s13065-022-00899-7

**Published:** 2022-11-22

**Authors:** Maryam Bigdelo, Firouzeh Nemati, Yalda Rangraz

**Affiliations:** 1 Department of Chemistry, Semnan University, Semnan, 35131-19111 Iran; 2 Semnan University, Semnan, Iran

**Keywords:** Organoselenium compounds, Heterogeneous mesoporous catalyst, SBA-15, Dehydration reaction, Nitrile derivatives

## Abstract

**Background:**

Here we report a new selenium-based heterogeneous catalyst, which was prepared from the immobilization of diphenyl diselenide on amine-functionalized Santa Barbara Amorphous-15 (SBA-15). The catalyst characterization study has been confirmed by different analysis methods including Fourier transform infrared spectroscopy (FT-IR), thermogravimetric analysis (TGA), energy-dispersive X-ray spectroscopy (EDX), X-ray diffraction patterns (XRD), field-emission scanning electron microscopy (FE-SEM), and Brunauer–Emmett–Teller (BET) surface area analysis.

**Results:**

The newly designed catalyst was successfully applied in the green dehydration reaction of oximes to corresponding nitriles in the presence of hydrogen peroxide/air.

To demonstrate the role of the catalyst in this study, the model reaction was also carried out in the absence of the catalyst and a trace yield of the relevant product was achieved.

**Conclusion:**

In this way, a series of nitrile derivatives were obtained with 72–96% yields, also, the catalyst could be separated easily and recycled for four consecutive runs with no obvious drop in catalytic activity.

## Introduction

Organoselenium compounds have been considered a research interest because of their effective role in many organic reactions for the production of important synthons and reagents. [1–4]. Selenium is a cheap and environment-friendly element, which can be metabolized in the body, so exhibits superior activity with less harmful toxic waste in a wide range of organic transformations as the catalyst [5]. One of the serious problems in chemical reactions is the separation of catalysts from the reaction medium. For this purpose, the boost of highly efficient heterogeneous catalysts with some competencies such as facile recovery, and low catalyst leaching is prime of importance.

One of the beneficial molecular frameworks is nitrile compounds, which are used as the main part of agrochemicals, pharmaceuticals, and fine chemicals [6, 7]. In addition, these compounds can be rendered for the preparation of a wide range of organic materials such as heterocycles, and the compounds containing carbonyl functional groups in green reaction approaches [8, 9].

In the past decades, several protocols including transition-metal heterogeneous catalysts have been reported for the synthesis of nitrile compounds [10, 11]. However, these methods suffer from costly heterogeneous catalyst systems, metal or non-metal chemical dehydration, and producing a large number of waste products [12]. Therefore, recent protocols with subtle and inexpensive reagents with the metal-free approach under greener and milder conditions are well desired.

One of the methods for the preparation of nitriles is dehydration of oximes, which has a good atom economy. Oxime is an organic compound produced by the reaction of an aldehyde or a ketone compound, and hydroxylamine contains a polar group and can participate in several organic chemical reactions [13]. Nitriles have attracted significant attention from chemists due to their remarkable synthetic properties as important intermediates for the synthesis of carboxylic acids, esters, aldehydes, ketones, amides, and amines. Furthermore, nitriles are very useful starting materials for the production of agrochemicals, pharmaceuticals, and biologically active compounds such as thiazoles, tetrazoles, 2-oxazolines, oxazoles, triazoles, diarylimidazoles, and benzamidines [14–17].

However, Yu et al. reported a practical method for the synthesis of organonitrile compounds from dehydration of oximes in moderate to good yields using diaryl diselenides as the catalysts [10]. The catalyst could be reused for four to six cycles without the inevitable loss of its catalytic activity. The main drawback of this protocol was the laborious separation of the catalyst.

To overcome these disadvantages, the heterogenization of a homogeneous catalyst onto solid supports could be excitant. Recently, the development of new heterogeneous catalysts according to the concept of “Green sustainable chemistry” is highly needed. It has been estimated that almost all commercially produced chemical products utilize heterogeneous catalysts [18]. So, large attention has been devoted to the demand for material as solid support for the preparation of heterogeneous catalysts. Among them, ordered mesoporous silica materials, which are silicates obtained by hydrothermal synthesis and a liquid templating mechanism are prime of importance [19]. SBA-15 mesoporous silica is one of the popular mesoporous silicas that utilizes for the design and development of heterogeneous catalysts. It has wide applicability in the design of materials and applied catalysts due to the comparatively thicker walls resulting in higher mechanical and thermal durability [20]. Functionalization of the surface of SBA-15 with organic or inorganic functional groups [21–24] results in new chemical and physical properties [25].

Considering the aforementioned and in the continuation of our recent efforts on the design and application of organoselenium heterogeneous catalysts in organic reactions [26–31], especially our first attempt at the oxidation of aldehydes to carboxylic acids [32], herein, we wish to report, the metal-free reaction for dehydration of oximes for producing nitrile compounds. In the present strategy, the organoselenium anchoring onto inorganic mesoporous support as a heterogeneous catalyst was employed in the presence of H_2_O_2_/air as the clean oxidant. The catalyst was easily recycled with simple filtration and reused without obvious deactivation.

## Experimental

### Materials

Aryloximes were prepared according to the reported method in the literature [33]. Arylaldehdyes, hydroxylamine hydrochloride, sodium borohydride, *p*-aminobenzoic acid, *N–N’-*dicyclohexylcarbodiimide (DCC), (3-aminopropyl) triethoxysilane (APTES), selenium element, and common reagents were obtained from Sigma-Aldrich company and used as received. All the solvents were obtained from Amertatco and used without further purification.

The procedure for steps of the synthesis of catalyst SBA-15-Se)_2_.

### SBA-15-NH_2_ synthesis

The inorganic mesoporous silica (SBA-15) was synthesized according to the reported procedure [34], and then modified using (3-aminopropyl) triethoxysilane (APTES) as follows: In a 100 mL flask, 3 g of mesoporous SBA-15 silica was refluxed with 3 mL of 3-(3-aminopropyl)triethoxysilane (12.8 mmol) in the 10 mL of toluene for 18 h. After that, the resulting solid product is filtered and dried in an oven at 90 °C during the night [35].

### 4,4′-diselanediyldibenzoic acid synthesis

The 4,4′-diselanediyldibenzoic acid was synthesized according to our previous report [32].

### Immobilization of organic selenium ligand on SBA-15; Synthesis of SBA-15-Se)_2_

0.15 g of 4,4′-diselanediyldibenzoic acid, 0.1 g of *N, N*-dicyclohexyl carbamide (DCC), and 0.1 mL of triethylamine were added to approximately 10–15 mL of degassed CH_2_Cl_2_ and was stirred at 0 °C under nitrogen atmosphere for 30 min. Thereupon, a suspension of 0.1 g of mesoporous silica in 10 mL of dichloromethane was added to that mixture and magnetically stirred for an additional 1 h. It was then stirred at room temperature for 16 h. Subsequently, the reaction mixture was stirred at 40 °C for another 3 h, the solid was filtered and washed with dichloromethane, and dried at 60 °C.

### General procedure for preparation of arylnitriles

Oxime (1 mmol), acetonitrile (2 mL), hydrogen peroxide 0.025 mL, and SBA-15-Se)_2_ (0.02 g) as catalyst were mixed thoroughly. The reaction mixture was stirred under airflow at 65 °C and the progress of the reaction was followed by thin-layer chromatography (TLC). After completion of the reaction, the mixture was cooled to room temperature. Hot ethanol was added to the reaction mixture, and the catalyst was separated from the reaction mixture by centrifugation. After the separation of the catalyst, the goal products were purified by recrystallization from hot ethanol or by plate chromatography using n-hexane/ethyl acetate (9/1 ratio) as the mobile phase and silica gel 60 HF_254_ as the stationary phase. All the products were known and identified by the appearance of the absorption spectral band related to the cyanide (CN) functional group in FTIR spectra and comparison of melting points with those reported in the literature or handbooks.

## Results and discussion

### Fabrication and characterization of SBA-15-Se)_2_

**Fig. 1:**
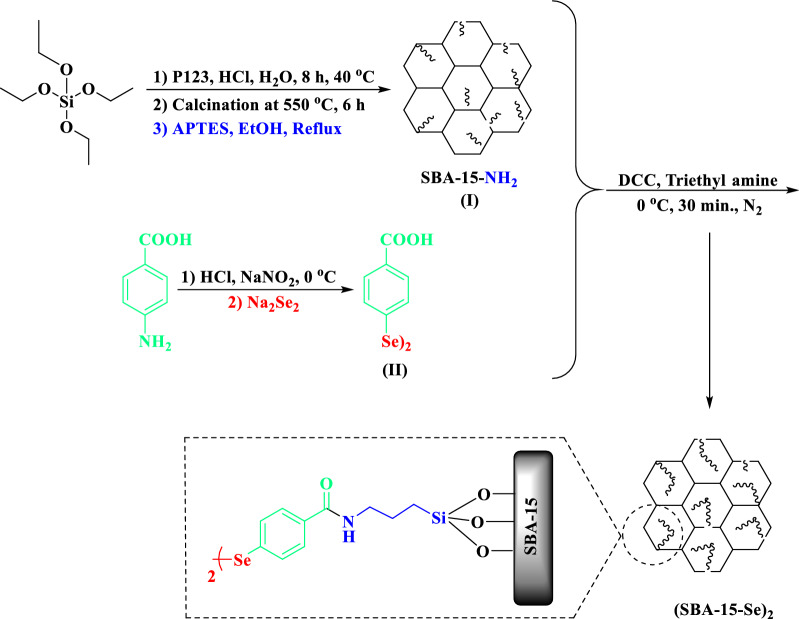
The steps of preparation of SBA-15-Se)_2_

4,4′-diselanediyldibenzoic acid (II) was obtained from the azotization reaction of para-aminobenzoic acid in the presence of Na_2_Se_2_. The obtained product was treated with aminopropyl modified SBA-15 in appropriate conditions to afford the organoselenium grafted onto inorganic mesoporous support as depicted in Fig. 1 in two steps.

**Fig. 2:**
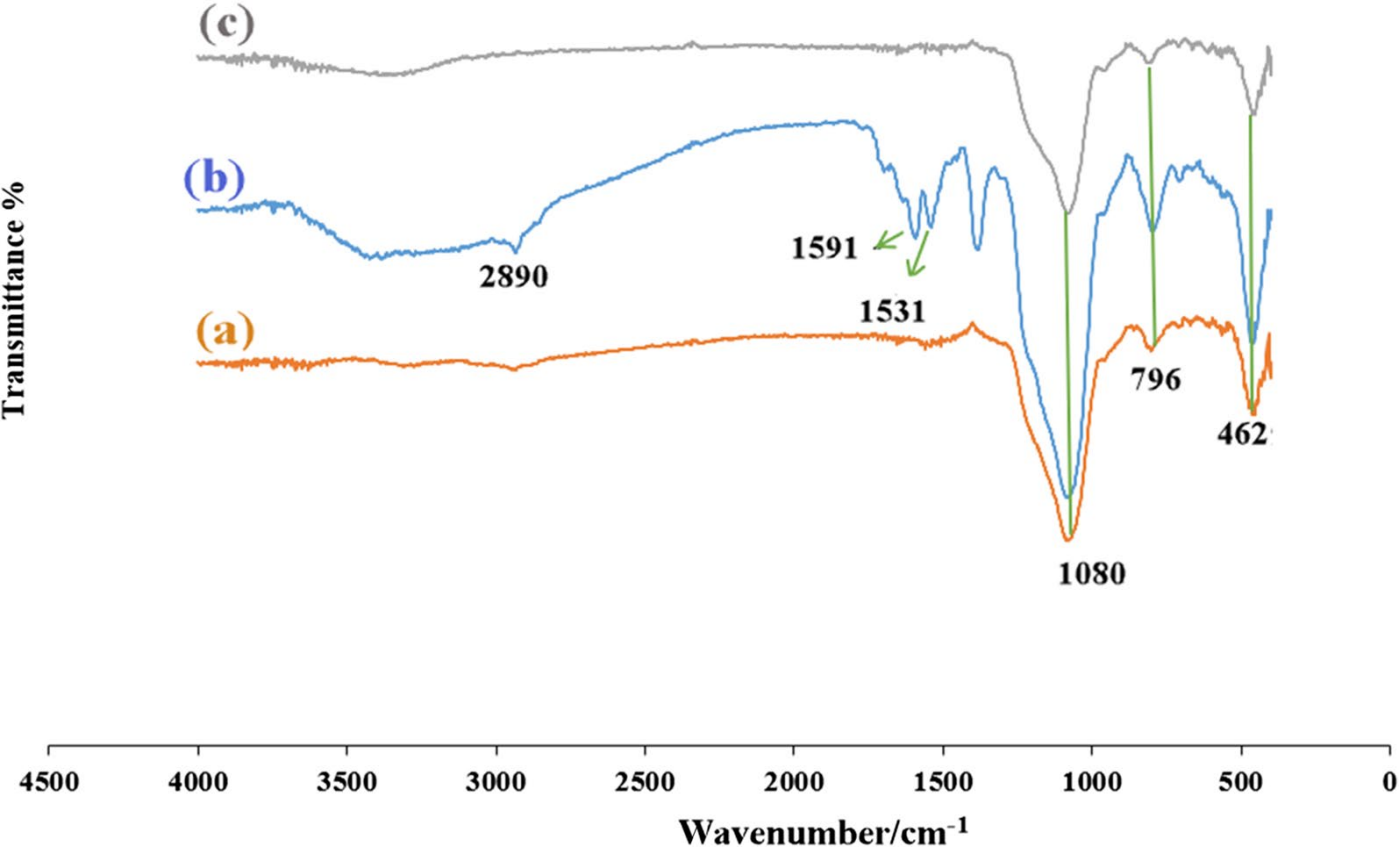
FTIR spectra of **a** SBA-15, **b** SBA-15-NH_2_ and **c** SBA-15-Se)_2_

Figure 2 contains three FT-IR spectra that represent SBA-15, SBA-15-NH_2,_ and SBA-15-Se)_2_. The absorption bands at 1080, 796, and 462 cm^−1^, which were clearly observed can be attributed to’ Si–O-Si, Si–O, and Si–O-Si vibrations in SBA-15, respectively. The vibrations of Si–OH groups appeared at 950 and 3380 cm^−1^ [35]. The presence of a weak peak at 2890 cm^−1^ is supposed to be C-H stretching vibration, and it suggests that almost all surfactant was ousted (Fig. 2a). After modification of SBA-15 with APTES, the vibration peaks of -CH_2_ and -CH groups were detected at 2890 cm^−1^. Meanwhile, two distinct absorption bands of NH_2_ were observed at 1591 and 1531 cm^−1^, which approved the successful grafting of amine groups to the surface of inorganic mesoporous support (Fig. 2b) [36]. The spectrum of SBA-15-Se)_2_ (Fig. 2c) displayed weak absorption bands due to the placement of functional groups in the holes of SBA-15. Therefore, the results above proved that the organoselenium segment was successfully functionalized.

**Fig. 3:**
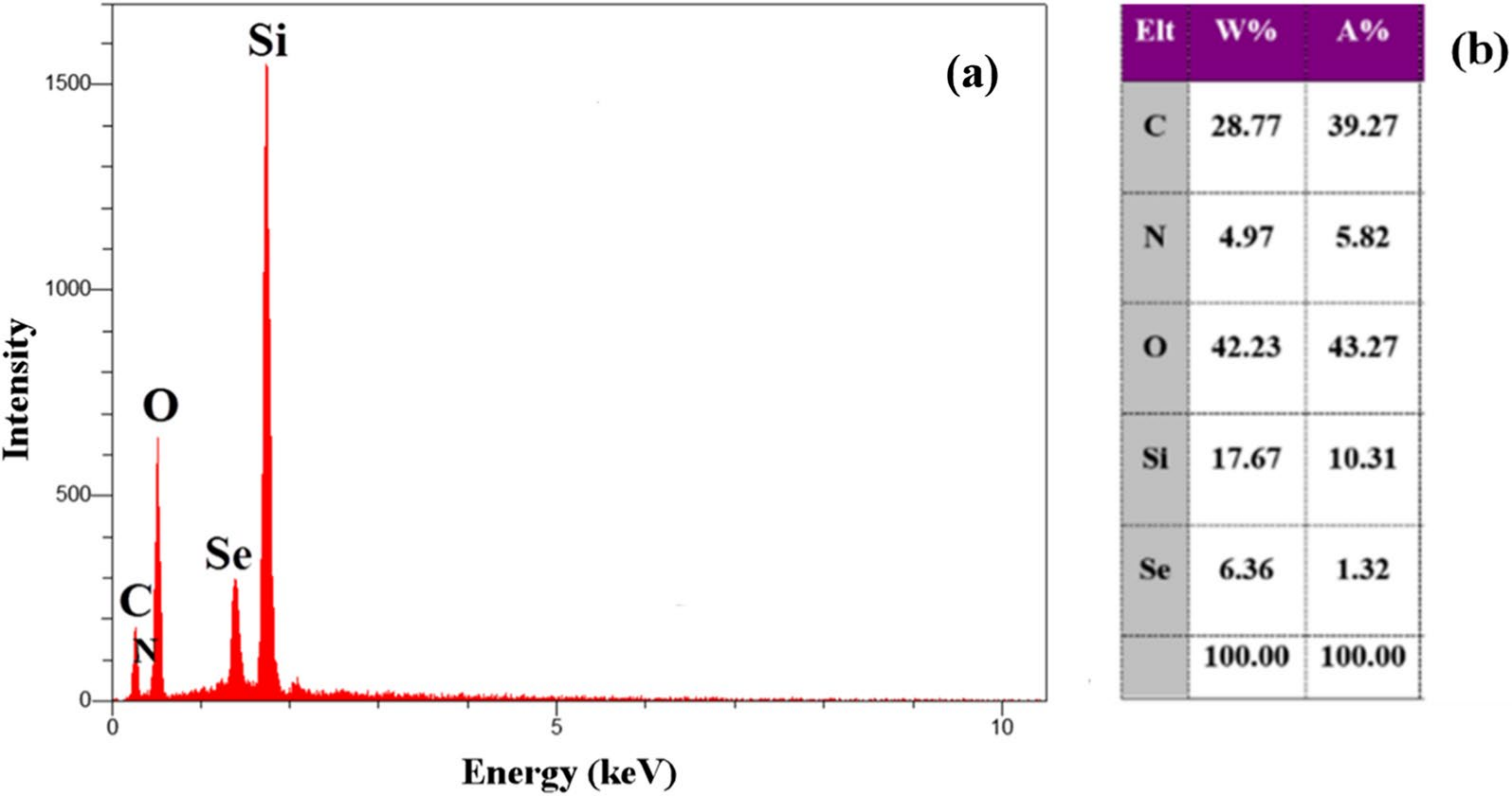
EDX spectrum of SBA-15-Se)_2_ **a** and compositional analysis of SBA-15-Se)_2_ **b**

**Fig. 4:**
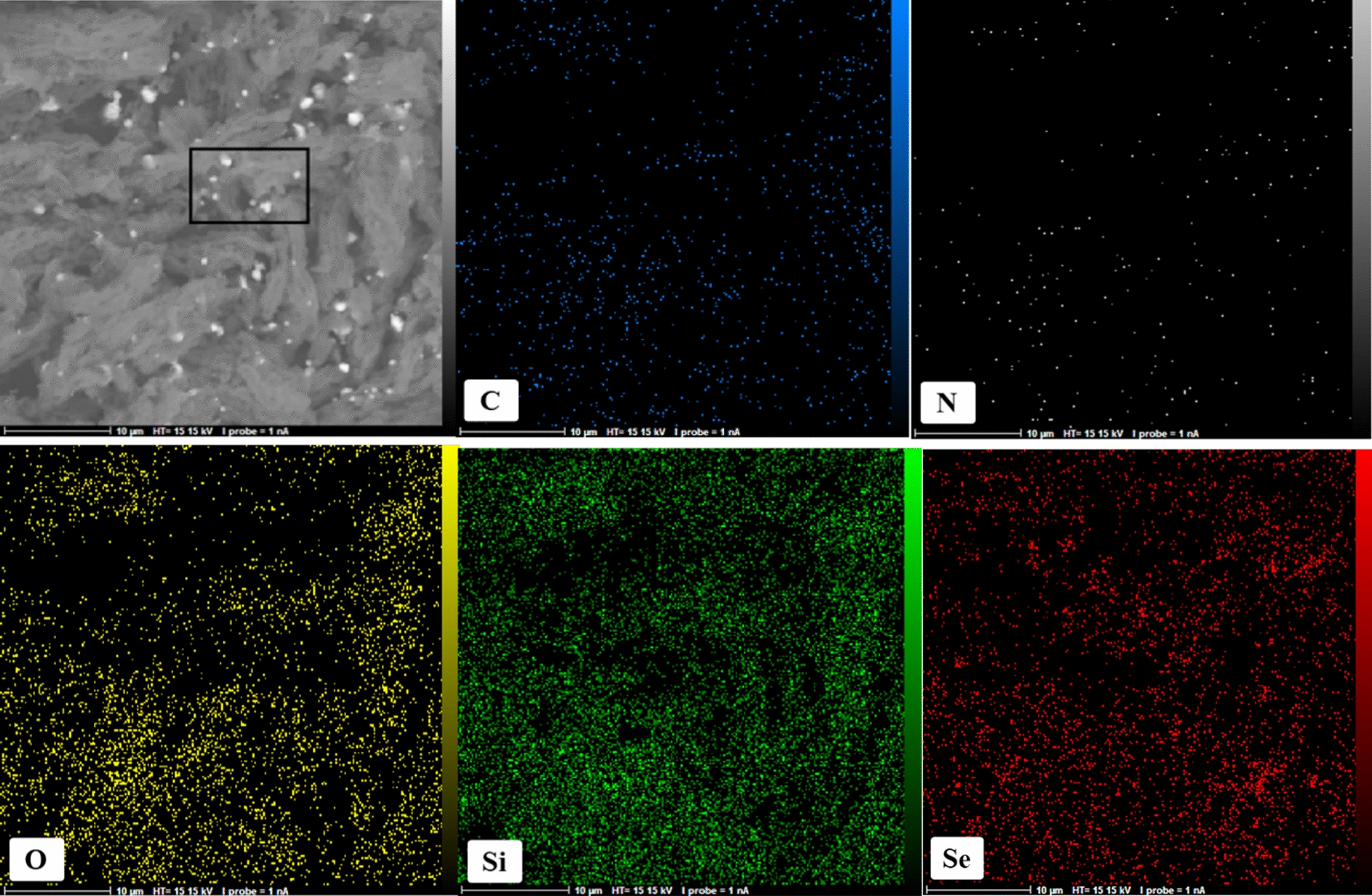
Elemental mapping analysis of SBA-15-Se)_2_

Meanwhile, to further verify the element composition of the final catalyst, energy dispersive x-ray spectroscopy was performed. Figure 3 depicts the results of the EDX spectrum obtained from sample of SBA-15-Se)_2_. The EDX spectrum of SBA-15-Se)_2_ shows the presence of carbon, nitrogen, oxygen, silicon, and selenium atoms, thus confirming the successful functionalization of SBA-15 using organoselenium compounds. 6.36 wt% selenium was extended in the SBA-15-Se)_2_ as displayed in the Fig. 3b. Furthermore, the selected area was subjected to elemental mapping patterns, which shows the homogeneous dispersal of all the component elements in the SBA-15-Se)_2_ (Fig. 4).

**Fig. 5:**
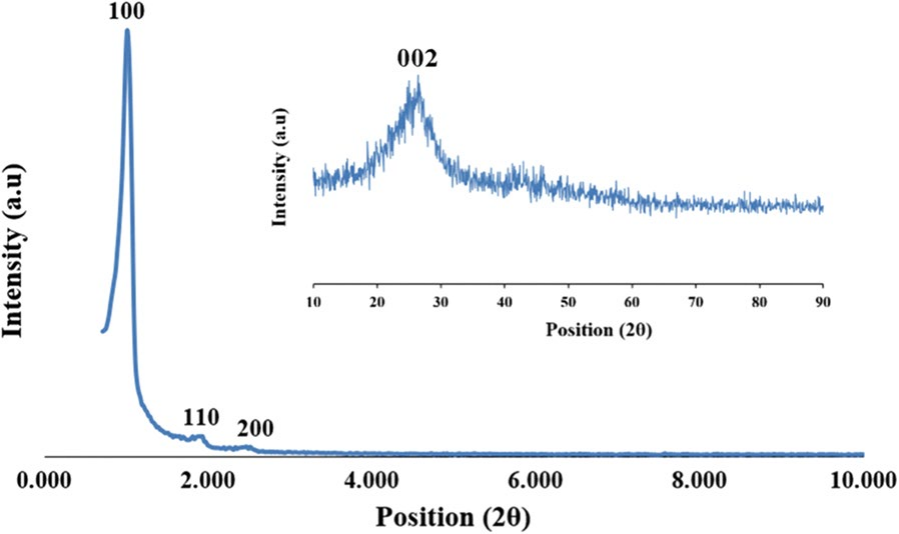
XRD patterns of the SBA-15-Se)_2_

The low-angle XRD pattern of SBA-15-Se)_2_ in the range of 0.4–12° was represented in Fig. 5. The hexagonal symmetry of three-dimensional inorganic mesoporous support (SBA-15) was confirmed with three typical diffraction peaks of (1 0 0), (1 1 0), and (2 0 0), which indicates the hexagonal structure of SBA-15 was maintained after functionalized with organoselenium moiety [37]. Only a certain decrease in the intensity of peaks (in comparison with literature) mentions that the mesoporous ordering reduced when it was treated with organic groups. However, the indicating peak pattern demonstrates that the inside of the mesoporous channels was grafted mainly with selenium organic functions [36]. In addition, the broad band between 2*θ* = 20–30° illustrates the amorphous nature of the organic matter, which was encapsulated in the channel of SBA-15 (inset of Fig. 5).

**Fig. 6:**
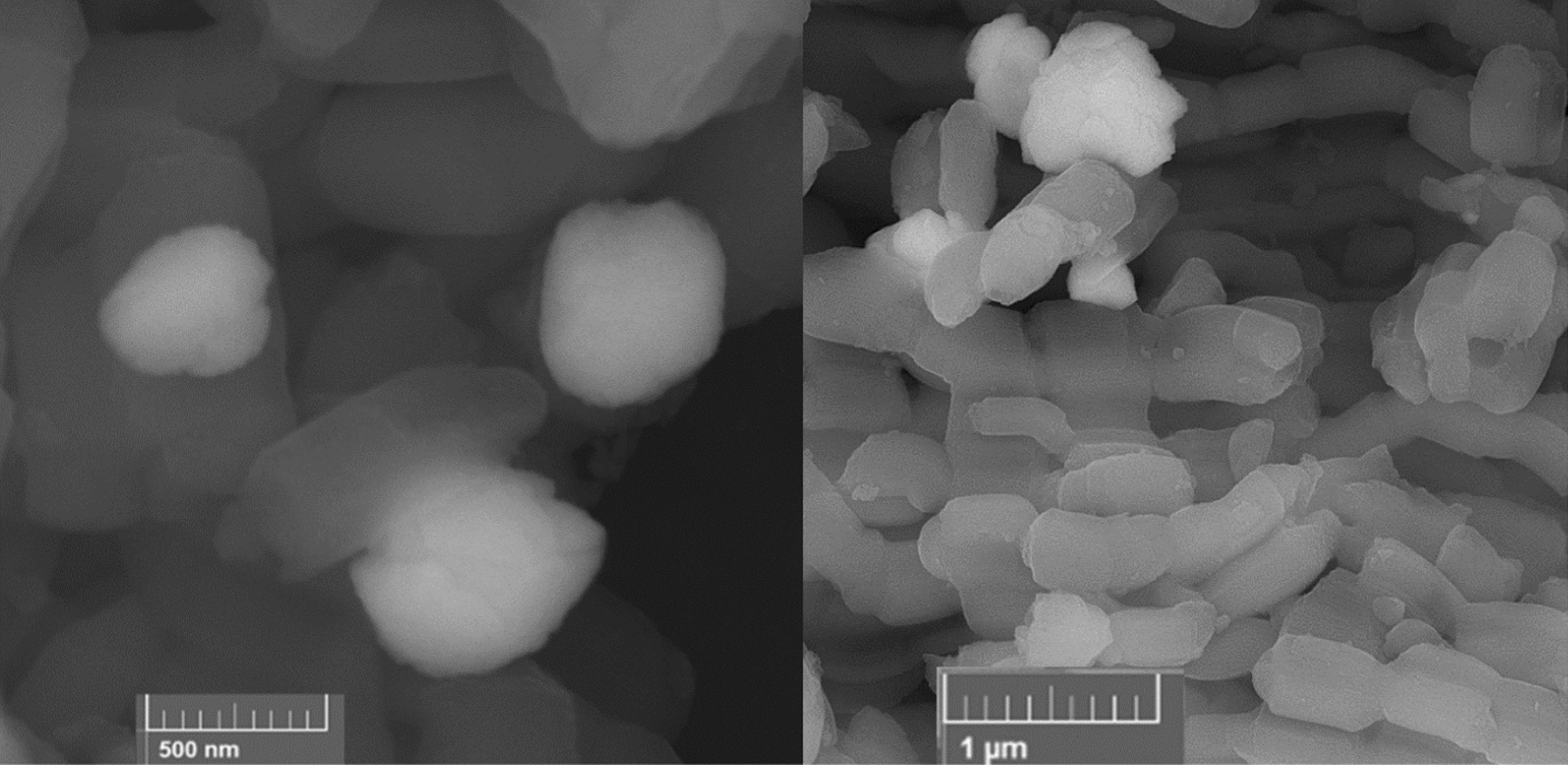
The FESEM images of SBA-15-SeS)_2_ in different magnifications

As shown in the FE-SEM images of the SBA-15-Se)_2_ sample, the presence of good-ordered particles that array regularity was seen. The particles are ranging from 900 to 1300 nm with uniform distribution. In addition, the ordered mesoporous structure of SBA-15 support was similar to that of reported ones, and remained unchanged after the grafting of the organoselenium part present in both the surface and channels of SBA-15 as depicted in Fig. 6 [38]. The bright spots are selenium, which appears bright in an SEM image compared to lighter elements, such as silicon because more backscattered electrons are emitted from the sample surface [39].

**Fig. 7:**
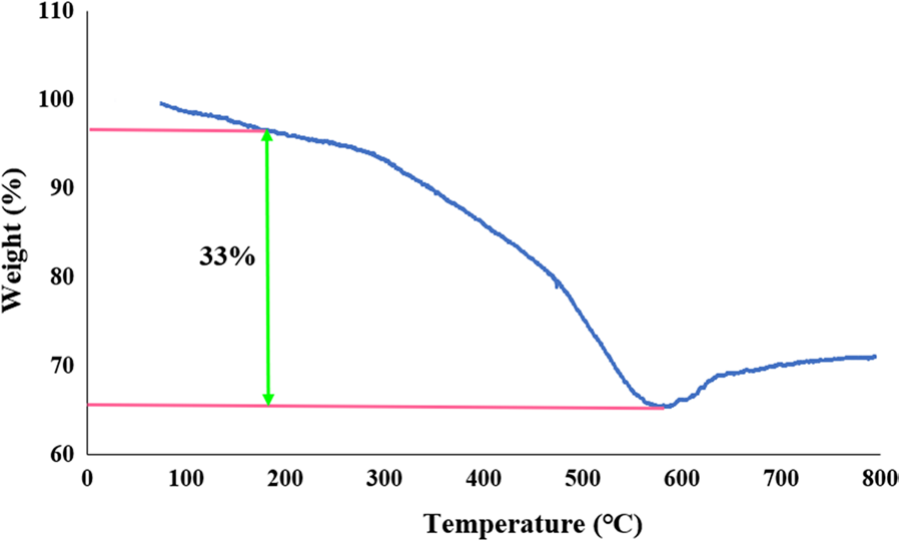
TGA curve of SBA-15-Se)_2_

Figure 7 shows the thermal gravimetric analysis (TGA) curve of SBA-15-Se)_2_, which shows weight loss due to decomposition on heating. The first minor amount of mass loss (about 5–6%) below 200 °C is related to the departure of physicosorbed solvent molecules [27]. The second breakdown in the range of 200–550 °C (nearly 33%) corresponds to the decomposition of organoselenium functional groups [32]. In continue, a tangible increase in weight is.

observed at 600–800 °C, which may be due to the re-adsorption of the combusted materials [40]. The estimated amount of grafted organoselenium compounds, which can be obtained by the residue of SBA-15 in the sample, is 1.23 mmol g^−1^ of inorganic mesoporous support. Therefore, the thermogravimetric analysis supported that adequate organoselenium functional groups have been chemically linked on the SBA-15 surface.

**Fig. 8:**
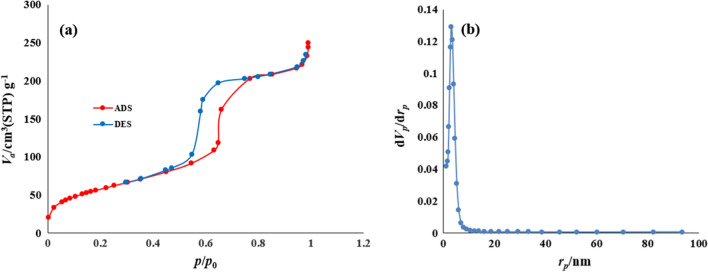
**a** Adsorption/desorption isotherm and **b** pore size distribution curve of SBA-15-Se)_2_

The isotherm of the SBA-15-Se)_2_ could be categorized as a type of IV with an H_1_ hysteresis loop, by mesoporous materials [41] (Fig. 8). The as-prepared catalyst exhibited a lower Brunauer–Emmett–Teller surface area (S_BET_, 212.06 m^2^/g) in comparison with unmodified SBA-15 (Table 1). The decrease in S_BET_ can be attributed to the incorporation of at least part of the organoselenium groups inside the mesoporous. Evidently, the rapid N_2_ adsorption–desorption rate in P/P_0_ between 0.68 and 0.8 demonstrated a typical capillary adsorption phenomenon with uniform pore distribution. Apparently, with increasing the P/P_0_ to 0.8–1.00, N_2_ adsorption rarely changed, which suggested the adsorption had reached saturation [35].

**Table 1:** Summary of BET measurements

Sample	S_BET_^b^ (m^2^g^−1^)	V_T_^c^ (cm^3^g^−1^)	D_p_^d^ (nm)
SBA-15	510.02	0.77	7.86
SBA-15-Se)_2_	212.06	0.38	7.21

^a^N_2_ sorption isotherms data at− 196 °C

^b^Specific surface area from BET

^c^Total volume

^d^Pore size

### Investigation of the catalytic activity of SBA-15-Se)_2_ in the synthesis of nitriles

#### Optimization of reaction conditions

To optimize the conditions, the conversion of 4-chlorobenzaldehyde oxime to 4-chlorobenzonitrile was initially chosen as the pattern transformation. So, on heating in 1 mL water at 65 °C, 1 mmol oxime with 0.25 mL H_2_O_2_ (2.45 mmol) in the presence of 0.02 g SBA-15-Se)_2_ as the catalyst was mixed under airflow. The corresponding nitrile product was generated in trace yield (Table 2, entry 6). The reaction was performed in other protic solvents such as MeOH and EtOH but led to low yield as well (Table 2 entries 7 and 8). Using acetonitrile as a solvent gave higher yields to 95% and it was the best solvent choice. The non-polar solvents were also tested, but they were unfavorable for this reaction (Table 2, entries 9 and 10).

**Table 2:** Optimization of reaction conditions of conversion of 4-chlorobenzaldehyde oxime to 4-chlorobenzonitrile^a^


Entry	Cat./g	Temp. (°C)	H_2_O_2_ (30%) mmol	Time (h)	Solvent	Yield (%)
1	None	65	2.45	10	Acetonitrile	Trace
2	0.01	65	2.45	10	Acetonitrile	78%
**3**	**0.02**	**65**	**2.45**	**10**	**Acetonitrile**	**95%**
4	0.03	65	2.45	10	Acetonitrile	95%
6	0.02	65	2.45	10	Water	Trace
7	0.02	65	2.45	10	EtOH	55
8	0.02	65	2.45	10	MeOH	55
9	0.02	65	2.45	10	Toluene	Trace
10	0.02	65	2.45	10	CHCl_3_	Trace
11	0.02	65	1.2	10	Acetonitrile	72
12	0.02	65	3.6	10	Acetonitrile	95
13	0.02	75	2.45	10	Acetonitrile	83

Bold values indicate the optimum condition

^a^Reaction condition: aldoxime (1 mmol), solvent 3 mL under airflow

To attain more transformation of the starting material, the temperature of the reaction was raised from 65 °C to 75 °C, but it was found that 65 °C was the superior temperature, which gives the product of the model reaction in 95% yield (Table 2, entry13). After determining the best solvent and setting the reaction temperature, the amount of H_2_O_2_ was also optimized. This revealed that the reduction of H_2_O_2_ dosage dejected the yield of the reaction (Table 2, entry 11) but using more than H_2_O_2_ did not prosper the product yield as anticipated (Table 2, entry 12). When no catalyst was added, the reaction gave a poor yield of the product (Table 2, entry 1).

#### Scope of reaction

Under the optimized case, means 0.02 g SBA-15-Se)_2_ (equals to 1.6 mol% Se) as heterogeneous organocatalyst, 2.45 mmol H_2_O_2_, 3 mL acetonitrile as a solvent, and airflow, the series of aryloximes was engaged to the synthesize of the corresponding arylnitriles. A series of aldoximes including electron-enriched or electron-donation substrates gave desired products in moderate to high yields (Table 3, entries 1, 2, 8, and 9). The substrates with electron-deficient groups were obviously preferred better and arylnitriles produced in high yields (Table 3, entries 3–7). Therewith, this protocol could be applied to the bulky and heterocycle-containing substrate, and desired product occurred an acceptable yield (Table 3, entries 10 and 11).

**Table 3:** The catalyst performance of SBA-15-Se)_2_ for various substrates

Entry	Substrate	Product	Time (h)	Yield%
1			7	90
2			8	87
3			4	96
4			7	95
5			4	96
6			10	96
7			10	95
8			10	86
9			8	72
10			12	75
11			11	83

^a^Reaction conditions: oxime (1 mmol), SBA-15-Se)_2_ (0.02 g), H_2_O_2_ (2.45 mmol), acetonitrile (2 mL), 65 °C

#### Plausible reaction mechanism

**Fig. 9:**
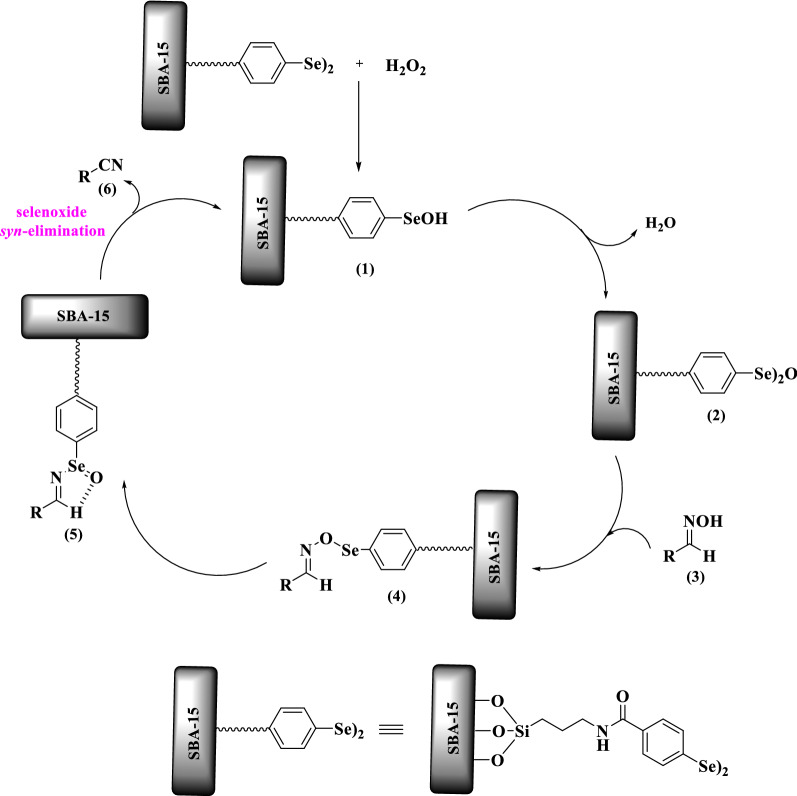
Proposed mechanism for dehydration of aldoxime catalyzed by SBA-15-Se)_2_

A proposed mechanism for the aldoxime dehydration reaction is displayed in Fig. 9 based on the previous literature [3, 10, 42]. Initially, selenenic acid (1) is generated through diselenide oxidation using hydrogen peroxide, which is then converted into the relevant selenenic anhydride (2). In the next step, condensation of aldoxime (3) with selenenic anhydride (2) gives intermediate (4). The rearrangement of (4) lids to the intramolecular hydrogen bond-stabilized intermediate (5), which decomposes to product nitrile (6) and regenerated the catalytic species (1) using a selenoxide syn-elimination process.

#### Reusability of SBA-15-Se)2

**Fig. 10:**
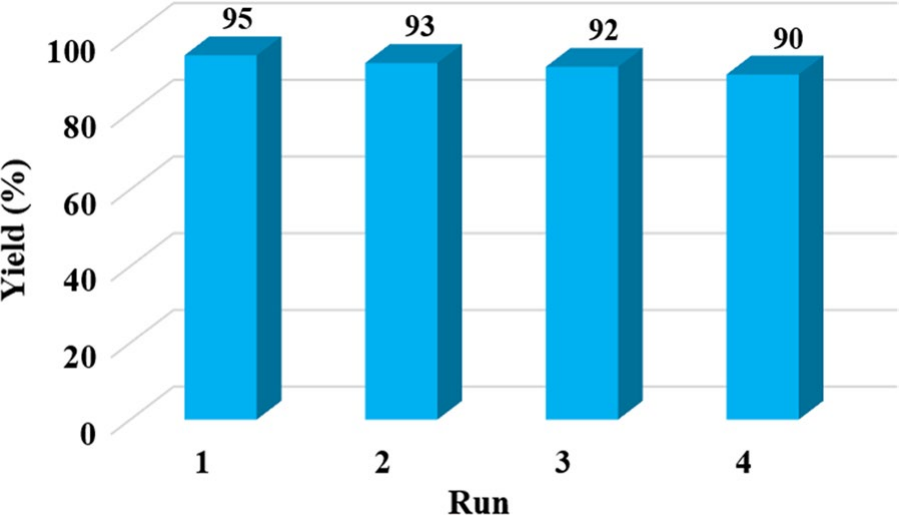
Diagram of percent yield results based on repeat test cycles for recycling of the catalyst

Eventually, to validate the durability and industrial applicability of SBA-15-Se)_2_, recycling tests were carried out under optimized conditions. After completion of the reaction, the catalyst was recollected using a centrifuge, washed thoroughly with acetone and ethanol, and then dried at 60 °C for other cycles. Figure 10 displays that the SBA-15-Se)_2_ can be reutilized for at least four consecutive runs with no considerable decrease in catalytic activity.

## Conclusion

In summary, we have demonstrated a facilitated strategy to prepare a heterogeneous selenium-based catalyst via immobilization of organic selenium on SBA-15 modified with APTES. The as-synthesized catalyst was used as an efficient catalyst for the conversion of aldoximes to arylnitriles using hydrogen peroxide and air. Various characterization techniques such as FTIR, XRD, FESEM, EDX-Mapping and BET were performed to confirm the formation of as-obtained catalyst. Based on its catalytic performance, this heterogeneous selenium-based catalyst with high recycling efficiency has huge potential to be explored in environmental remediation. The excellent recyclability, mild reaction condition, using green oxidant, metal free catalyst, and durability of the catalyst are highlights of this transformation. No reference standards or controls are indicated for comparison in regards to the performance of the new-catalyst. This study endeavors to add to the existing library of catalysts that can potentially be exploited in the future for industrial conversions of oximes to nitrile products in perhaps more efficient ways.

## Data Availability

All data generated or analyzed during this study are included in this published article.

## Abbreviations

SBA-15: Santa barbara amorphous-15
FT-IR: Fourier transform infrared spectroscopy
TGA: Thermogravimetric analysis
EDX: Energy-dispersive X-ray spectroscopy
XRD: X-ray diffraction
FE-SEM: Field emission scanning electron microscopy
BET: Brunauer emmett-teller
DCC: Dicyclohexylcarbodiimide
APTES: (3-Aminopropyl) triethoxysilane
H_2_O_2_: Hydrogen peroxide
TLC: Thin-layer chromatography
CH_2_Cl_2_: Dichloromethane
EtOH: Ethanol
MeOH: Methanol
CHCl_3_: Chloroform
